# Oxidative aging facilitates biological barrier penetration of polyethylene microplastics, amplifying systemic lipotoxicity in aquatic species

**DOI:** 10.1186/s12989-026-00689-2

**Published:** 2026-06-07

**Authors:** Yejin Kim, Hyerin Lee, Eunji Lee, Yun Hak Kim, Chang-Kyu Oh

**Affiliations:** 1 Department of Convergence Medical Sciences, School of Medicine, Pusan National University, Yangsan, 50612 Republic of Korea; 2 Department of Biochemistry, School of Medicine, Pusan National University, Yangsan, 50612 Republic of Korea; 3 Interdisciplinary Program of Genomic Science, Pusan National University, Yangsan, 50612 Republic of Korea; 4 Department of Biomedical Informatics, School of Medicine, Pusan National University, Yangsan, 50612 Republic of Korea; 5 Department of Anatomy, School of Medicine, Pusan National University, Yangsan, Republic of Korea; 6 Institute for Future Earth, Pusan National University, Busan, 46241 Republic of Korea

**Keywords:** Zebrafish, *Daphnia magna*, Microplastic, Oxidation, Lipid transport

## Abstract

**Background:**

Environmental aging processes, such as oxidation, can substantially modify the physicochemical properties and toxicity of microplastics (MPs). Nevertheless, most studies have focused on pristine MPs, overlooking aged forms that more accurately represent environmental exposure conditions. Understanding the toxicological consequences of oxidative aging is essential for realistic ecological risk assessment.

**Results:**

We investigated the toxicological effects of pristine polyethylene (PE) and oxidized polyethylene (OPE) microplastics using a dual-species aquatic model comprising *Daphnia magna* and zebrafish (Danio rerio) embryos. Physicochemical characterization revealed that OPE particles exhibited increased surface roughness, a more negative surface charge, and a higher proportion of oxygen-containing functional groups on the particle surface compared with PE. Exposure to OPE induced pronounced lipid accumulation and significantly reduced heart rate in both models. Transcriptomic analysis indicated that OPE downregulated key genes related to lipid transport and metabolism, including *mttp*, *apoea*, and *apobb*. These findings were further validated by quantitative PCR and Oil Red O staining. Notably, zebrafish embryos exposed to OPE displayed developmental impairment even with intact chorions, implying enhanced bioavailability and barrier penetration of oxidized particles.

**Conclusions:**

Our findings demonstrate that oxidative aging amplifies the biological toxicity of polyethylene microplastics by disrupting lipid metabolism and developmental processes. This study underscores the importance of considering environmentally aged MPs in ecological risk evaluations, as pristine particles may underestimate their actual hazard potential in aquatic ecosystems.

**Graphical Abstract:**

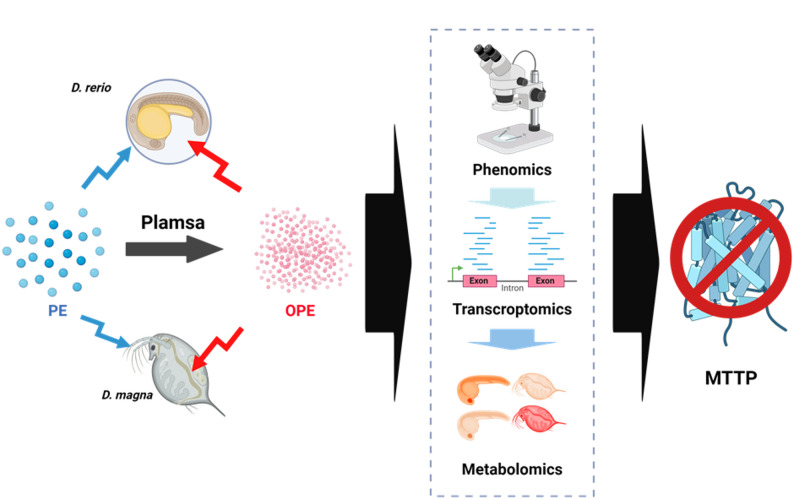

**Supplementary Information:**

The online version contains supplementary material available at 10.1186/s12989-026-00689-2.

## Introduction

Plastics have become indispensable in modern society, but their widespread use has led to the accumulation of microplastics (MPs) in terrestrial and aquatic environments [1, 2]. These MPs originate from plastic waste, industrial processes, and household products, with global plastic production exceeding 380 million tons annually [3, 4]. Among them, polyethylene (PE) is one of the most commonly detected types in the environment due to its durability and extensive use [5]. Many organisms, including aquatic and terrestrial species, inadvertently ingest microplastic particles, which can accumulate in their bodies and lead to both mechanical damage and chemical toxicity [6].

A growing number of studies have demonstrated that MPs exert multifaceted toxicity across biological systems. For example, exposure to PE microplastics impairs reproductive function in livestock and polystyrene (PS) particles at concentrations ranging from 0.01 to 1000 µg/mL induce behavioral alterations and inflammatory responses in mice [7]. MPs have also been shown to disrupt lipid metabolism [8], generate reactive oxygen species (ROS) that damage DNA [9], and induce inflammation, thereby compromising immune responses [10]. Our previous work similarly revealed that PE exposure induces cardiotoxicity in zebrafish embryos, even with an intact chorion [11]. Among these, disruption of lipid metabolism is particularly concerning as it can impair energy balance and systemic physiological functions. Together, these findings emphasize the multi-dimensional toxicological effects of MPs on both physical health and metabolic homeostasis [12–14]. In natural settings, MPs are subject to surface modification via environmental aging [15, 16]. While natural weathering involves a multi-factorial complex of processes, surface oxidation stands out as a critical chemical transformation that fundamentally alters particle surface reactivity [17–20]. Therefore, examining how specific oxidative functional groups dictate biological interactions is crucial to understanding the potential long-term risks of aged microplastics [21, 22]. Notably, oxidized polyethylene (OPE) fragments generated through advanced oxidation processes or photooxidation have demonstrated heightened toxicity in both Caco-2 intestinal cells and murine intestinal tissues [23].

These findings emphasize the importance of conducting toxicological studies on oxidation-associated surface changes, as oxidation-associated changes are often overlooked in studies that rely solely on pristine particles. Such oxidation-associated changes can be captured through physicochemical characterization and are expected to alter particle–biological interactions [16, 24]. Oxidized microplastics (OMPs) exhibit altered surface properties that can intensify biological interactions and toxic effects; therefore, it is important to compare pristine and oxidized particles in biologically relevant in vivo systems [23, 25, 26]. In particular, aquatic ecosystems serve as the primary sinks and exposure pathways for microplastics, making aquatic organisms highly sensitive indicators for assessing microplastic toxicity [27–30]. Furthermore, in vivo models enable an integrated evaluation of organism-level responses such as metabolic disturbance, oxidative stress, inflammation, and developmental abnormalities that cannot be fully captured by in vitro systems [31–33]. Based on this rationale, we considered that in vivo experiments using aquatic species are essential to elucidate the biological effects of pristine polyethylene (PE) and oxidized polyethylene (OPE) microplastics. Among in vivo models, *Daphnia magna* and zebrafish (*Danio rerio*) are widely used because of their environmental relevance and experimental utility [34–36]. These models are particularly valuable in developmental and molecular toxicology due to their transparency, genetic tractability, accessibility, and similarity to vertebrate systems [18, 37]. Zebrafish also enable the use of advanced techniques such as qPCR, phenotypic screening and even single-cell RNA sequencing, making them powerful models for investigating MP-induced lipid metabolic alterations [38, 39]. While the toxic effects of oxidized or aged microplastics have been increasingly reported [40, 41], less is known about how the specific structural shift from pristine to oxidized polyethylene affects vertebrate and invertebrate models at a mechanistic level. In this study, we sought to fill this gap by employing a highly controlled oxygen plasma treatment as an accelerated surface oxidation model. This approach allows us to eliminate confounding environmental variables (e.g., biofouling or co-contaminants) and isolate the pure toxicological impacts of surface-bound oxygen functional groups. By comparing PE and OPE using a dual-species in vivo model, this study provides a comprehensive framework for assessing OPE toxicity and offers new insights into how oxidation-associated surface modifications may influence the biological interactions and toxicological outcomes of polyethylene microplastics in aquatic organisms.

## Materials and methods

### Preparation of polyethylene microplastics

Polyethylene (PE) microplastic powder with a particle size range of 1–4 μm was purchased from CD Bioparticles (Shirley, New York, USA). The powder was dispersed in 1 mL of triple-distilled water (TDW) containing 0.01% Tween 20 in a glass vial. The suspension was sonicated to achieve a uniform dispersion and subsequently used for the exposure experiments.

### Preparation of oxidized polyethylene microplastics

Polyethylene (PE) powder was oxidized by exposure to a Clone4 plasma device (CIONE4, Femto Science Inc., Republic of Korea) operated at 80 kHz for 60 min. The resulting oxidized polyethylene (OPE) particles were then dispersed in 1 mL of triple-distilled water (TDW) containing 0.01% Tween 20 to ensure homogeneous suspension. Before each experiment, the OPE suspension was sonicated to achieve uniform dispersion and used immediately for exposure experiments.

### Characterization of polyethylene and oxidized polyethylene

The sizes of the PE and OPE particles were measured using a Zetasizer Nano ZSP (Malvern Panalytical, United Kingdom), and their surface charges were also determined with the same device. Surface etching and morphological changes in the PE were examined using scanning electron microscopy (SEM) with a Hitachi FE-SEM S-4700 (Hitachi, Japan). Additionally, Fourier-transform infrared spectroscopy (FTIR) measurements were conducted using the iS50 model (Thermo Fisher, United States) on the samples in their powder form. X-ray photoelectron spectroscopy (XPS) was performed using an AXIS SUPRA system (Kratos Analytical) equipped with an Al Kα X-ray source (hν = 1486.6 eV). The analysis was carried out under a vacuum better than 1 × 10⁻⁸ Torr at 15 kV and 15 mA. Binding energy was calibrated using the C 1s peak at 284.8 eV, and the spectra were analyzed using CasaXPS software. The XPS spectra were plotted using Origin.

### Maintenance of *Daphnia magna*

Adult *Daphnia magna* were obtained from the Environmental Engineering Laboratory at Pusan National University and cultured under controlled laboratory conditions. They were housed in 2-liter glass beakers containing M4 medium, in accordance with OECD Guideline 202. Cultures were maintained at 22 °C with a 16:8 h light-dark photoperiod in a temperature-controlled incubator. The culture medium was refreshed twice weekly to ensure water quality and organism health. *D. magna* were fed daily with chlorella at a concentration of 5 × 10⁷ cells/mL. The chlorella cultures were grown in a medium supplemented with calcium nitrate, magnesium sulfate, and nutrient solutions (3 Brothers Hydroponics, Republic of Korea). Prior to feeding, 500 mL of chlorella culture was harvested by centrifugation at 500 × g and resuspended in fresh M4 medium.

### Exposure of *Daphnia magna* to microplastics

Juvenile *Daphnia magna* (0–24 h post-birth) were exposed in groups of five per vial, with each individual kept in 4 mL of M4 medium containing PE or OPE microplastics at specified concentrations. Exposures were conducted under static conditions at 22 °C with the same photoperiod as the maintenance phase. After 48 h, phenotypic and physiological assessments were performed. Heart rate was measured under an S9i stereomicroscope (Leica Microsystems, Wetzlar, Germany) by counting beats for 10 s and converting to beats per minute (bpm). At least 20 individuals were analyzed per treatment group. Immobilization was assessed according to OECD Test Guideline 202. After 48 h of exposure, individuals were considered immobilized if they failed to swim within 15 s following gentle agitation of the medium. The percentage of immobilized individuals was recorded for each group.

### Maintenance of zebrafish

Adult zebrafish (*Danio rerio*, AB strain) were maintained in an automated recirculating system (Techniplast, Italy) at 28.5 ± 0.5 °C. Water quality was controlled at pH 7.0 and conductivity of 1000 µS/cm. A photoperiod of 14 h light and 10 h dark was applied, and fish were fed GEMMA Micro 300 (Skretting, USA) twice daily. Fertilized eggs were obtained through natural spawning and cultured in E3 embryo medium (14.61 g NaCl, 1.99 g MgSO₄, 1.83 g CaCl₂·2 H₂O, and 0.63 g KCl per liter of deionized water) at 28.5 °C. All experimental procedures were approved by the Institutional Animal Care and Use Committee (IACUC) of Pusan National University (approval number: PNU-2023-0359).

### Exposure of zebrafish to microplastics

For exposure experiments, PE powder (20 mg) was dispersed in 1 mL of triple-distilled water (TDW) containing 0.01% Tween^®^ 20 (Sigma-Aldrich) and sonicated for 10 min to achieve a homogeneous suspension. Zebrafish embryos were selected at 6 h post-fertilization (hpf) and exposed to PE and OPE microplastics at concentrations of 0.1, 1, and 10 mg/L in E3 medium until 48 hpf. All exposures were conducted under semi-static conditions, with daily renewal of the test medium at 28.5 °C.

### RNA sequencing

Zebrafish embryos at 48 hpf were euthanized using Tricaine (MS-222, Sigma-Aldrich, St. Louis, MO, USA) at a concentration of 2 g/L in TDW. After anesthesia, the embryos were washed three times with E3 medium and then stored at -80 °C overnight. The frozen embryos were then homogenized in TRIzol, and the homogenized samples were sent to Rokit Genomics (Seoul, South Korea) for further analysis.

### Quantitative PCR (qPCR)

Total RNA was extracted from 48 hpf zebrafish embryos using TRIzol reagent (Invitrogen, Waltham, MA, USA), and cDNA was synthesized using the SuperScript IV First-Strand Synthesis System (Thermo Fisher Scientific, Waltham, MA, USA). Quantitative PCR was performed using SYBR Green Master Mix (Thermo Fisher Scientific, MA, USA) on a real-time PCR system. All reactions were conducted in triplicate. Gene expression levels were normalized to the housekeeping gene *β-actin*, and relative expression was calculated using the 2^–ΔΔCt method.

**Table:** 

Gene	Forward Primers	Reverse Primers
*apobb1*	CAA GGC AGT CGC AGA TTA CA	CAC GCT TCT GTA TTG GAG CA
*apoc2*	TTG TTG CTT TCC TTG CAC TG	CAG CTG GTC TTG AAA GAT GC
*apoa4b.1*	GCA ACA CAA ACT GCA GAG GA	TGG CTC TTC ATG TTG TCA GC
*apobb.2*	TCT GCA TGC TTT TCA ACT GG	TCA ACA CTG ATG GTG GCA TT
*apoeb*	CAT GGT GCA AAA CAT CAA GG	CAC GTC ATC TGC ATT CTG CT
*apoa1a*	CCA ATT TGT TCC AGG CTG AT	TTG GAT CGG AGA TCC TCA AC
*apoea*	GAG GGA CAA CAT CAA GGC CA	GAT CCT TCG CCT CCT CCA TG
*cetp*	TCC TCC CCA GTG ATC AAA TC	CCA GAG ATA CTG CGC ACA AA
*apoba*	TCA GAA TGT CGA AGC ACC TG	CGG GAG AGC TTT GTG AAG TC
*mttp*	CTC AGC TGG TGG ATG CAG TA	ATC TCT GTG CTG CCG ATC TT

### Nile red staining of *Daphnia magna*

Juvenile *Daphnia magna* (0–24 h post-birth) were immersed in 4% paraformaldehyde (PFA) and fixed overnight at 4 °C. After fixation, specimens were thoroughly rinsed with phosphate-buffered saline (PBS) to remove any remaining fixative. Subsequently, the daphnids were exposed to a Nile Red working solution (1 mg/L in PBS) for 30 min at room temperature in the dark to avoid photobleaching. Following staining, the samples were washed three times in PBS to eliminate excess dye. The stained individuals were mounted on glass slides and visualized under a fluorescence stereomicroscope (SteREO Discovery.V8, Zeiss, Oberkochen, Germany) equipped with the appropriate filter sets.

### Nile red staining of zebrafish embryos

Zebrafish embryos at 48hpf were fixed overnight in 4% paraformaldehyde at 4 °C. Post-fixation, embryos were gently washed several times with PBS to ensure complete removal of fixative residues. For lipid visualization, the samples were incubated in a Nile Red solution (1 mg/L prepared in PBS) for 30 min at RT, with protection from light throughout the process. After staining, three sequential PBS washes were performed to remove unbound dye. Imaging was conducted using a fluorescence stereomicroscope (SteREO Discovery.V8, Zeiss, Oberkochen, Germany).

### Statistical analysis of zebrafish embryo data

All quantitative data obtained from zebrafish embryo experiments were statistically analyzed using GraphPad Prism (v.5.0; GraphPad Software, San Diego, CA, USA). An unpaired two-tailed Student’s t-test was used to evaluate differences between two groups. Statistical significance was considered at *p* < 0.05.

### Protein-protein interaction (PPI) analysis using STRING

Protein–protein interaction (PPI) analysis was performed using the STRING database (version 12.0; https://string-db.org). A list of differentially expressed genes was uploaded, and interactions were retrieved for Danio rerio. All available evidence sources (experimental data, databases, text mining, co-expression, neighborhood, gene fusion, and co-occurrence) were included. The minimum required interaction score was set to 0.700 (high confidence). Disconnected nodes were excluded from the final network visualization. The resulting interaction network was downloaded and further examined to identify functional clusters and hub proteins.

### Quantification and differential expression analysis

All computational analyses were performed in R (v.4.4.1). To begin, gene expression was quantified using Salmon, and the gene-level count matrices were prepared for downstream analysis using tximport (v.1.26.1). Identification of differentially expressed genes (DEGs) was accomplished with DESeq2 (v.1.38.3), using FPKM values as input. A significance cutoff was established at an adjusted p-value < 0.05. The log2 fold change estimates from this analysis were subsequently refined using the shrinkage estimator from apeglm (v.1.28.0).

### Pathway and gene ontology enrichment

The functional roles of significant DEGs were investigated through an enrichment analysis. We employed the clusterProfiler package (v.4.6.2) and the org.Dr.eg.db (v.3.16.0) annotation resource to perform Gene Ontology (GO) analysis. This procedure identified the biological pathways that were most significantly over-represented within the set of differentially expressed genes.

### Data visualization

Data visualization was performed using standard R packages. Three-dimensional principal component analysis (3D PCA) plots, volcano plots, Gene Ontology (GO) enrichment plots, heatmaps, and Venn diagrams were generated with ggplot2 (v3.5.1), pheatmap (v1.0.12), plotly (v4.10.4), VennDiagram (v1.7.3), and related tools to illustrate sample clustering, differential gene expression, functional enrichment, gene set overlap and gene expression patterns associated with GO terms.

### Data analysis using *Daphnia magna* database

The GSE149738 dataset (NCBI GEO) was analyzed using computational methods consistent with zebrafish analysis, with the following additions:

rtracklayer (v1.66.0) for *Daphnia magna* genome annotation processing.

ashr (v2.2-63) for enhanced precision in log₂ fold-change estimation.

## Results

### Characterization of polyethylene (PE) and oxidized polyethylene (OPE)

**Fig. 1:**
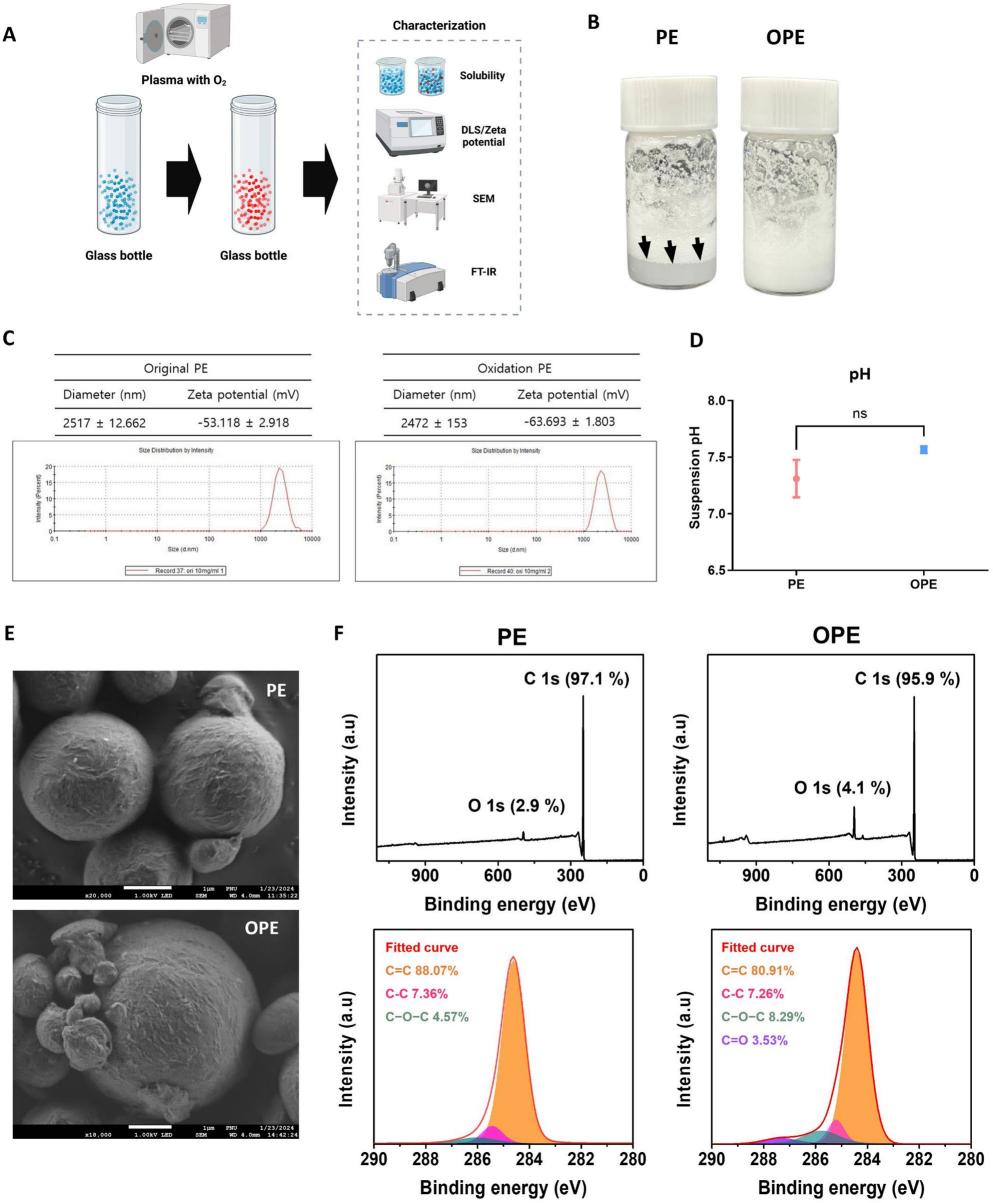
Characterization of pristine polyethylene (PE) and oxidized polyethylene (OPE). **A** Schematic diagram showing oxidation of pristine polyethylene (PE) particles using oxygen plasma and the subsequent characterization steps. **B** Suspension images of PE and OPE particles in water. (Black arrows indicate sedimented aggregates) **C** DLS and zeta potential analysis of PE and OPE particles in aqueous dispersion. **D** Measurement of suspension pH in PE and OPE under the same conditions used for zeta potential analysis. **E** SEM images showing surface morphology of dried PE and OPE particles. **F** Survey spectra showing increased surface oxygen content in OPE compared with PE. Deconvoluted C 1s spectra showing an increased proportion of oxygen-containing functional groups in OPE

To evaluate the physicochemical alterations induced by oxidation, pristine polyethylene (PE) particles were subjected to plasma oxidation to generate oxidized polyethylene (OPE) (Fig. 1A). The physicochemical properties of the particles were characterized using solubility analysis, dynamic light scattering (DLS), zeta potential measurement, scanning electron microscopy (SEM), and Fourier-transform infrared spectroscopy (FTIR).

Visual inspection revealed that OPE particles exhibited a more homogeneous dispersion compared to PE, suggesting reduced particle aggregation after oxidation (Fig. 1B). DLS analysis showed no significant difference in the mean particle size between the two groups; the average diameter of PE was 2517 ± 12.662 nm, while that of OPE was 2472 ± 153 nm, indicating that oxidation had minimal impact on the average particle size (Fig. 1C). However, the substantially larger standard deviation in the OPE group suggests greater heterogeneity in particle size distribution following oxidation. Furthermore, the zeta potential became more negative after oxidation, shifting from − 53.118 ± 2.918 mV for PE to − 63.693 ± 1.803 mV for OPE, reflecting an increase in surface charge density. To determine whether the difference in zeta potential was influenced by pH, the pH of PE and OPE suspensions was measured under the same experimental conditions used for zeta potential analysis. PE and OPE showed similar mean pH values of 7.3 and 7.6, respectively (Fig. 1D), indicating that pH differences between the two groups were negligible. Therefore, the altered zeta potential of OPE is unlikely to be attributable to pH differences and is more likely associated with oxidation-induced changes in particle surface characteristics. SEM imaging revealed the presence of surface fragments on OPE particles. While PE particles maintained a relatively smooth and spherical morphology, OPE particles displayed small, sharp, and irregular surface features, suggesting surface erosion and morphological alteration following plasma treatment (Fig. 1E). To further assess surface oxidation, XPS analysis was performed to compare the surface elemental composition of PE and OPE. The atomic concentration of O 1s increased from 2.9% in PE to 4.1% in OPE, whereas that of C 1s decreased from 97.1% to 95.9%, indicating increased surface oxygen content after plasma treatment (Fig. 1F). Deconvolution of the C 1s spectra further demonstrated that OPE contained a greater proportion of oxygen-containing functional groups than PE. Specifically, PE was composed of 88.07% C = C, 7.36% C–C, and 4.57% C–O–C, with no detectable C = O component, whereas OPE showed 80.91% C = C, 7.26% C–C, 8.29% C–O–C, and 3.53% C = O (Fig. 1G). FTIR analysis showed no marked spectral differences between PE and OPE, and both particles retained the characteristic absorption bands of polyethylene (Supplementary Figure S1). These results indicate that plasma oxidation altered the surface chemical composition of PE, while the overall polyethylene backbone remained preserved.

### Accumulation and physiological effects of PE and OPE in *Daphnia magna*

**Fig. 2:**
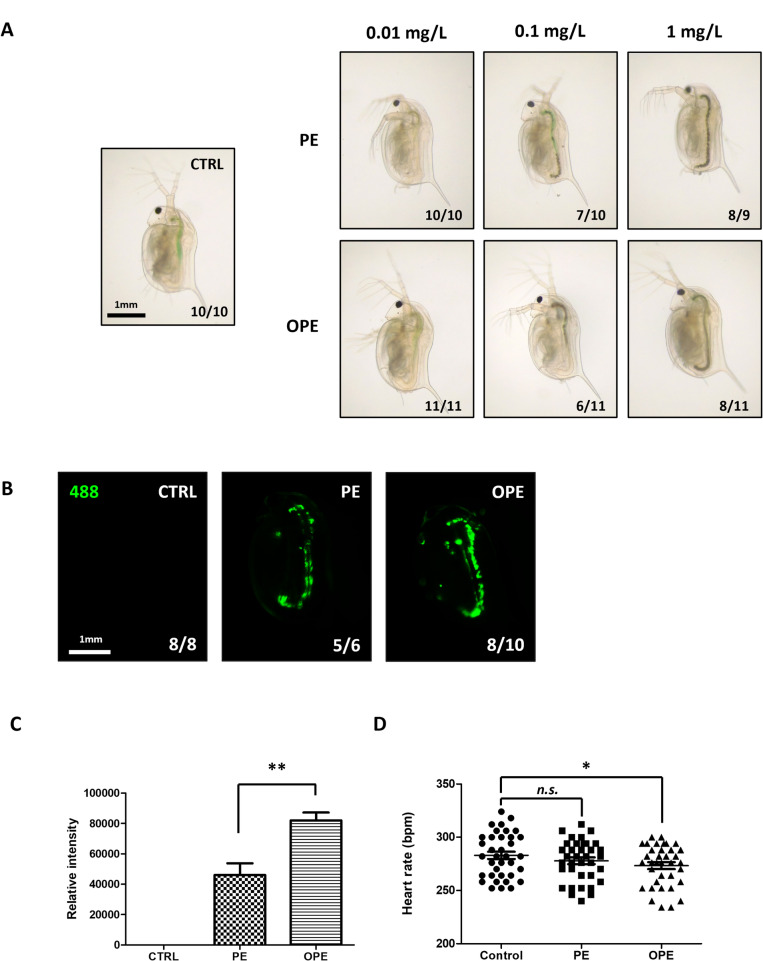
Accumulation and physiological effects of PE and OPE in *Daphnia magna*. **A** Representative images of *Daphnia magna* exposed to PE and OPE microplastics (0.01–1 mg/L). **B** Fluorescence images showing GI tract accumulation of 10–20 μm fluorescent PE microplastics (1 mg/L). **C** Quantification of fluorescence intensity. (*p* < 0.01). **D** Heart rate measurements in juvenile *Daphnia magna*. (*p* < 0.05)

Juvenile individuals of *Daphnia magna* were selected and exposed to PE and OPE microplastics at concentrations ranging from 0.01 to 1 mg/L for 2 days following an acute exposure protocol. Microplastic particles accumulated in the gastrointestinal tract (GI) after exposure (Fig. 2A). Fluorescent PE microplastics (10–20 μm, 1 mg/L) were localized along the gastrointestinal tract (Fig. 2B). Fluorescence intensity was significantly higher in the OPE group than in the PE and control groups (Fig. 2C). After the 2-day exposure, heart rate measurements in the exposed individuals revealed a significant decrease in the OPE group compared to both the PE and control groups (Fig. 2D).

### Transcriptomic and phenotypic alterations in lipid metabolism induced by OPE exposure

**Fig. 3:**
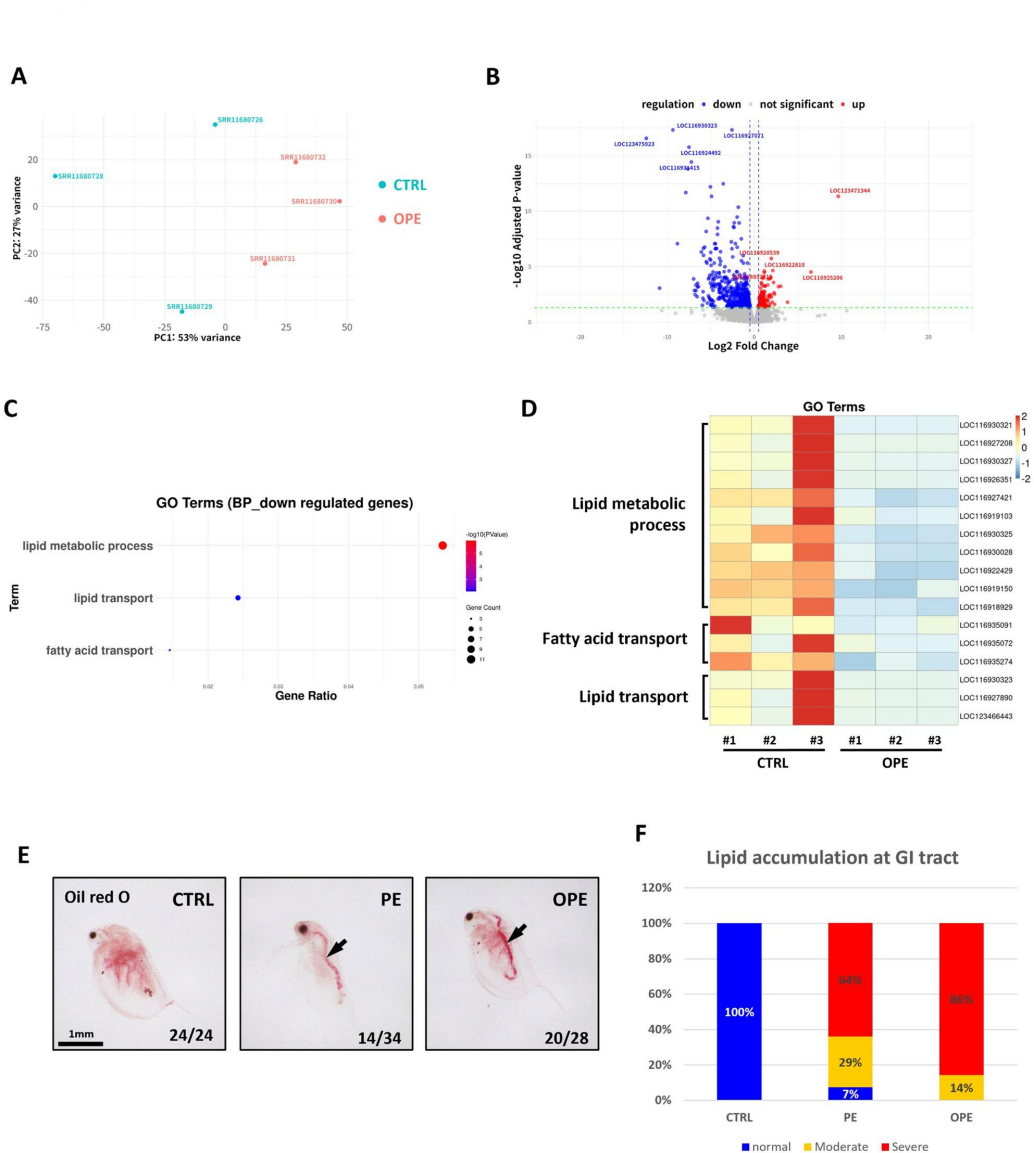
Transcriptomic disruption of lipid metabolism in*Daphnia magna*after OPE exposure.** A** Principal component analysis (PCA) plot showing separation between the control (CTRL) and OPE groups based on global gene expression. PC1 and PC2 explain 53% and 27% of total variance, respectively. **B** Volcano plot of differentially expressed genes. Red dots indicate upregulated genes, blue indicate downregulated genes, and gray indicate non-significant changes. **C** GO enrichment analysis of downregulated genes, showing significant terms related to lipid metabolism, lipid transport, and fatty acid transport. **D** Heatmap displaying expression patterns of genes associated with lipid-related GO terms in CTRL and OPE groups

To investigate the molecular effects of OPE exposure on lipid metabolism, RNA sequencing was performed. Principal component analysis (PCA) showed a clear separation between the control and OPE groups, indicating distinct gene expression profiles (Fig. 3A). Differential expression analysis revealed a large number of significantly downregulated genes in the OPE group, showing a clear trend of transcriptional suppression compared to the control and PE groups (Fig. 3B). Gene Ontology (GO) enrichment of the downregulated genes showed significant enrichment in “lipid metabolic process,” “lipid transport,” and “fatty acid transport,” suggesting that OPE broadly perturbs lipid-related biological pathways (Fig. 3C). A heatmap of representative genes further confirmed downregulation of lipid metabolism–related transcripts in the OPE group (Fig. 3D).

Phenotypic validation with Oil Red O staining revealed increased lipid accumulation in the GI tract of the OPE group (Fig. 3E). Partial staining was also observed in the PE group, whereas no staining was detected in the control group (Fig. 3F).

### OPE exposure induces developmental and physiological alterations in zebrafish embryos

**Fig. 4:**
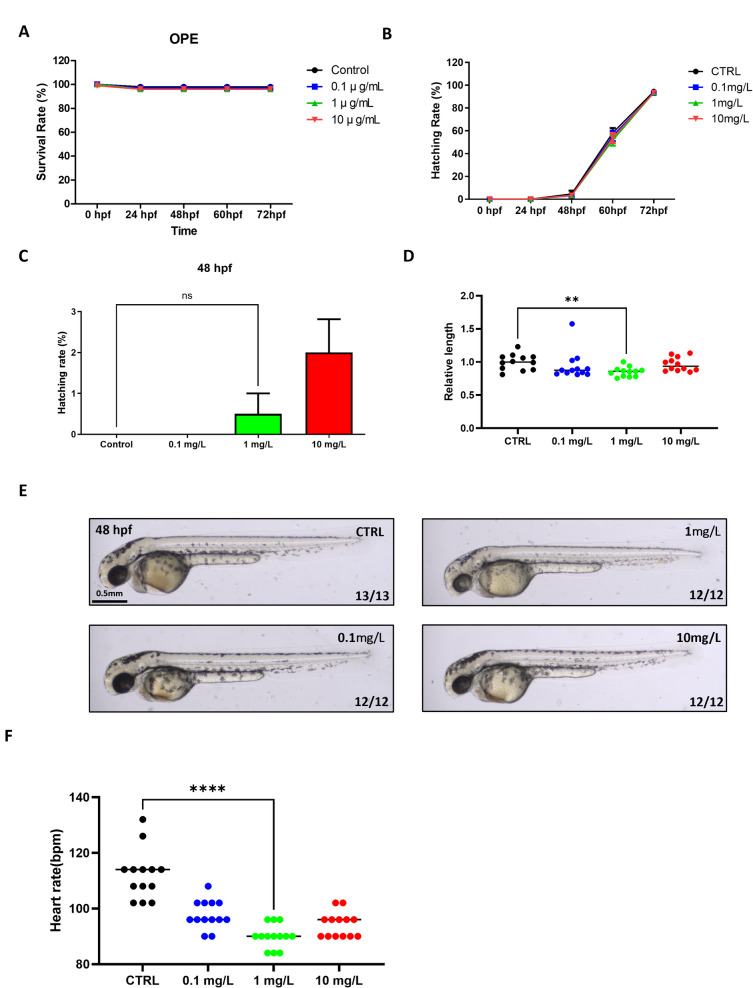
Developmental and physiological effects of OPE exposure in zebrafish embryos. **A** Survival rate of zebrafish embryos exposed to PE and OPE (0.1, 1, and 10 mg/L) until 72 h post-fertilization (hpf). **B**–**C** Hatching rate monitored from 0–72 hpf and at 48 hpf under each exposure condition. **D** Relative body length of embryos measured at 48 hpf. Significant reduction was observed in the 0.1 mg/L OPE group (*p* < 0.01). **E** Representative brightfield images of larvae showing reduced body length. **F** Heart rate measurement at 48 hpf. All OPE-exposed groups showed significantly reduced heart rate compared to control (*** *p* < 0.0001)

To investigate whether similar phenotypic effects observed in *Daphnia magna* also occur in other aquatic models, zebrafish embryos were used to assess the developmental toxicity of OPE. Embryos were exposed to various concentrations (0.1, 1, and 10 mg/L) from 0 to 72 hpf. Survival rates remained unchanged across all groups, including the control and both PE- and OPE-exposed groups (Fig. 4A). Hatching rates also showed no significant differences up to 72 hpf (Fig. 4B). However, OPE exposure showed a dose-dependent trend toward increased hatching, although the difference was not statistically significant at 48 hpf (Fig. 4C). Body length measurements at 48 hpf revealed a significant reduction in relative length at 0.1 mg/L compared to the control, whereas no further reduction was observed at higher concentrations (Fig. 4D). Representative images of larvae confirmed reduced body size in the OPE-exposed groups (Fig. 4E). Heart rate analysis demonstrated a clear and significant decrease in all OPE-treated groups compared to the control (Fig. 4F).

### OPE exposure disrupts lipid transport and metabolism in zebrafish embryos

**Fig. 5:**
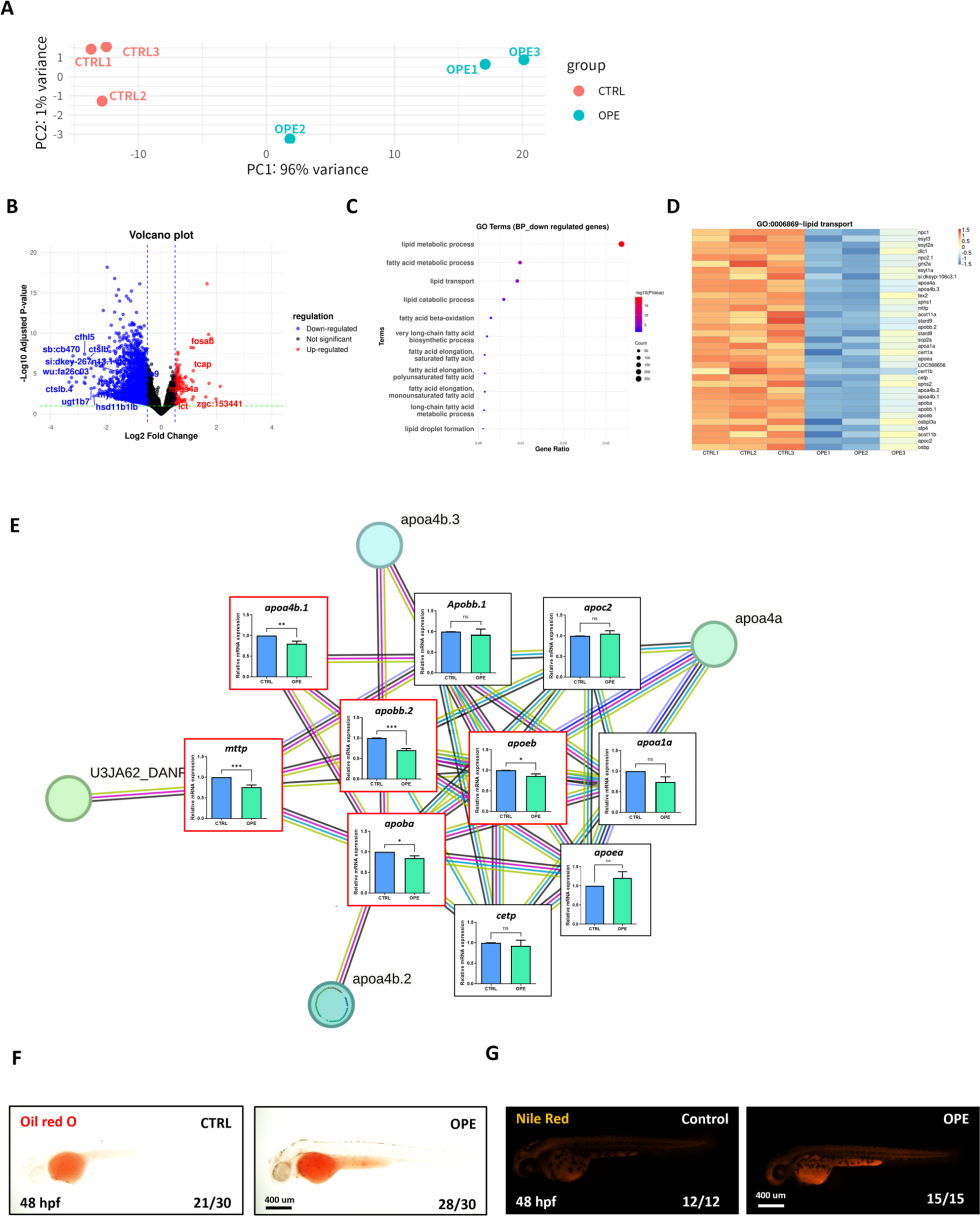
Disruption of lipid metabolism pathways by OPE exposure in zebrafish embryos.** A** PCA plot showing separation between CTRL and OPE groups. PC1 and PC2 account for 96% and 1% of variance, respectively. **B** Volcano plot of DEGs between CTRL and OPE. Red dots indicate upregulated genes, blue indicate downregulated genes, and black dots indicate non-significant genes. **C** GO enrichment analysis of downregulated genes showing terms related to lipid metabolic process, fatty acid metabolism, and lipid transport. **D** Heatmap showing expression of lipid transport-related genes (GO:0006869) across samples. **E** Gene interaction network and expression comparison of lipid transport-related genes. Nodes represent genes, and bars show expression changes between groups. Red borders indicate significant changes

To investigate whether the patterns observed in *Daphnia magna* were also observed in zebrafish embryos, RNA-seq analysis was conducted to evaluate the effect of OPE exposure on lipid-related pathways. Principal component analysis (PCA) revealed a clear separation between the OPE and control groups, highlighting distinct transcriptional profiles associated with OPE exposure (Fig. 5A). Volcano plot analysis showed a large number of significantly downregulated genes in the OPE group (Fig. 5B). Gene Ontology enrichment analysis of downregulated genes revealed significant enrichment in “lipid metabolic process,” “fatty acid metabolic process,” and “lipid transport” (Fig. 5C). A heatmap of genes within the GO term “lipid transport” further confirmed their downregulation in the OPE group (Fig. 5D). Protein–protein interaction (PPI) network analysis identified key genes involved in lipid metabolism that were significantly downregulated, including *apoa4a*,* apoa4b.1*,* apoa4b.2*,* apobb.1*,* apobb.2*,* apoea*,* apoeb*,* mttp*, and *cetp*. Validation using qPCR confirmed the transcriptomic trends for these genes (Fig. 5E). Phenotypic validation using Oil Red O staining showed increased lipid accumulation in the OPE group compared to control (Fig. 5F). Nile Red staining further confirmed elevated lipid deposition in the OPE group at 48 hpf. Notably, lipids appeared to accumulate within the yolk, suggesting impaired mobilization of yolk-derived lipids to developing tissues (Fig. 5G).

### Oxidized polyethylene induces transcriptional suppression of lipid metabolism–related genes

**Fig. 6:**
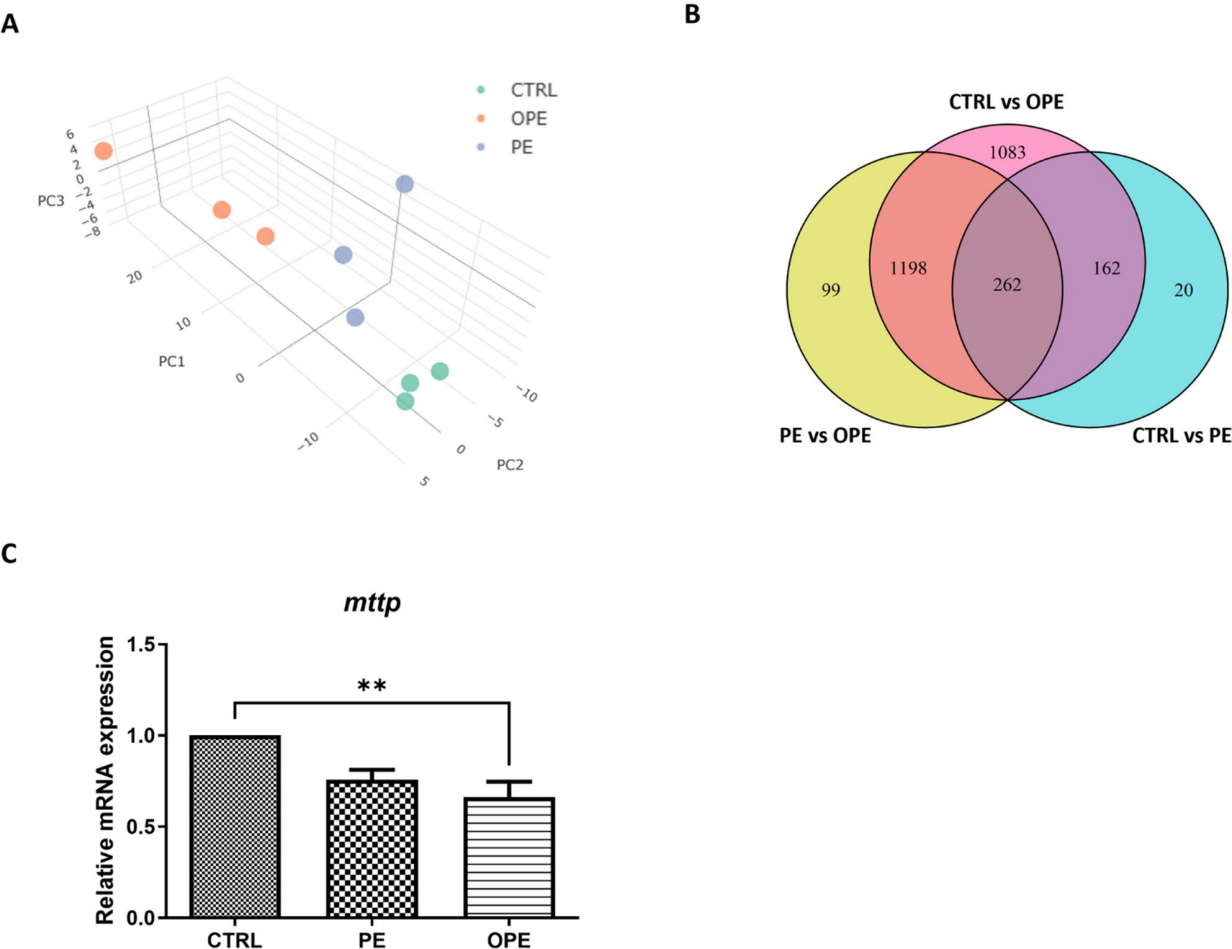
Oxidation of polyethylene enhances transcriptomic toxicity in lipid metabolism. **A** Principal component analysis (PCA) showing clear separation among control (CTRL), polyethylene (PE), and oxidized polyethylene (OPE) groups, with OPE exhibiting the greatest transcriptomic divergence. **B** Quantification of downregulated differentially expressed genes (DEGs) in CTRL vs. PE and CTRL vs. OPE comparisons, showing a markedly higher number of suppressed genes in the OPE group. **C** Venn diagram illustrating the overlap and unique distribution of DEGs across three comparisons (CTRL vs. PE, PE vs. OPE, and CTRL vs. OPE), with 1,198 DEGs uniquely altered in OPE exposure. **D** Heatmap of lipid transport–related genes showing consistent downregulation in OPE-exposed samples compared to CTRL and PE groups. **E** qPCR validation of *mttp* expression demonstrating stepwise reduction from CTRL to PE and most prominently in OPE (**p < 0.01)

To compare the transcriptomic impact of pristine polyethylene (PE) and oxidized polyethylene (OPE), three-dimensional principal component analysis (PCA) was performed. This analysis revealed distinct clustering of the control, PE, and OPE groups, with OPE showing the greatest divergence in global gene expression profiles (Fig. 6A). Quantification of differentially expressed genes (DEGs) demonstrated that the number of downregulated genes was markedly higher in the CTRL vs. OPE comparison than in CTRL vs. PE (Fig. 6B). Venn diagram analysis further indicated that 1,198 DEGs were uniquely altered in the CTRL vs. OPE comparison, whereas only a small number of unique DEGs were observed in CTRL vs. PE and PE vs. OPE (Fig. 6C). Focusing on lipid transport–related pathways, heatmap analysis revealed consistent downregulation of key genes such as *mttp*,* esyt3*, and members of the apolipoprotein family in the OPE group (Fig. 6D). These findings were validated by quantitative PCR, which confirmed a stepwise decrease in *mttp* expression from control to PE, and most prominently in OPE (Fig. 6E).

## Discussion

In this study, we provide compelling evidence that controlled oxidative modification of polyethylene significantly enhances microplastic toxicity in both invertebrate (*Daphnia magna*) and vertebrate (*Danio rerio*) aquatic models. A primary mechanism underlying this heightened toxicity is the disruption of lipid homeostasis through transcriptional suppression of lipid transport genes, exemplified by microsomal triglyceride transfer protein (MTTP). Impaired lipid mobilization provides a mechanistic link to the observed phenotypes, including abnormal lipid accumulation in the yolk sac of zebrafish embryos and the gastrointestinal tract of *D. magna*, as well as developmental impairments such as reduced heart rate and growth inhibition.

A key contribution of this study is the direct comparison between pristine and oxidized polyethylene, addressing a critical gap in the literature where most studies have focused solely on pristine MPs and may therefore overlook the toxicological contribution of oxidation-associated surface modifications [42, 43]. Our physicochemical analyses revealed that plasma oxidation induced notable surface changes such as increased roughness and a more negative zeta potential, which likely enhanced particle reactivity and biological interactions. In addition, XPS analysis showed that OPE exhibited increased surface oxygen content and a higher proportion of oxygen-containing functional groups compared with PE, supporting the interpretation that plasma treatment induced surface oxidative modification rather than extensive bulk oxidation. These surface-localized changes may have contributed to the enhanced toxicity observed in vivo. These results are consistent with recent findings that oxidized or surface-modified MPs can exhibit greater toxic potency [16, 44]. The robustness of these findings is further supported by the dual-model design, which revealed consistent toxicological patterns across phylogenetically distant species [45]. By maintaining chorion integrity, this study improves ecological relevance compared to previous works that relied on dechorionation or excessively high exposure levels [46, 47]. Importantly, our observations challenge the assumption that the zebrafish chorion serves as a complete barrier to microplastics, suggesting that oxidatively modified polyethylene particles or smaller fragments may penetrate chorion pores and interact directly with embryonic tissues [11, 46, 48].

Compared with previous studies using dechorionated embryos or experimental designs in which chorion integrity was disrupted, the present study maintained an intact chorion throughout exposure [46, 47]. This interpretation is consistent with previous reports showing that smaller plastic particles, including nanoplastics of 30 and 100 nm, exhibit enhanced uptake and biological interaction in zebrafish embryos [48]. In contrast, our study employed polyethylene microplastics in the 1–4 μm range and exposed embryos at concentrations of 0.1, 1, and 10 mg/L for 2 days while maintaining chorion integrity throughout the exposure period. This design better approximates environmentally relevant exposure conditions and allowed us to assess phenotypic responses under a more realistic exposure scenario. In some previous studies, much higher PE-MP concentrations ranging from 6.2 to 100 mg/L were used to induce more overt developmental toxicity in zebrafish embryos. Therefore, although our experimental conditions were not intended to produce the most extreme toxicity reported in the literature, they provide a more ecologically relevant framework for evaluating whether oxidative surface modification itself enhances the biological activity of polyethylene microplastics [46, 49].

Furthermore, our study revealed intriguing developmental responses that warrant deeper consideration. We observed a dose-dependent trend toward accelerated hatching in OPE-exposed embryos, and while not statistically significant, this could indicate a sublethal stress response, as toxicants can sometimes induce premature hatching as an escape strategy. More strikingly, the analysis of body length presented a non-linear dose-response, with significant growth reduction only at the lowest OPE concentration (0.1 mg/L). Such non-monotonic responses could be attributed to the activation of potent compensatory or detoxification mechanisms at higher concentrations that mitigate the initial toxic insult, or alternatively, to particle agglomeration reducing bioavailability. These complex effects highlight the need to assess a wide range of concentrations in toxicological studies to fully understand the biological impact of microplastics.

To elucidate the molecular basis of these phenotypes, we conducted transcriptomic analyses, which revealed significant suppression of lipid transport–related genes in OPE-exposed aquatic models. Among the most strongly downregulated transcripts in zebrafish embryos was *mttp*, a gene critical for the assembly and secretion of very-low-density lipoproteins (VLDL) and chylomicrons, indicating a severe impairment of systemic lipid mobilization. Beyond *mttp*, apolipoprotein-related genes including *apoa1a*,* apoea*,* apobb.1/2*, and *esyt3* were consistently suppressed. Apolipoprotein genes such as *apoa1a*,* apoea*, and *apobb* paralogs are involved in lipoprotein formation, cholesterol transport, and yolk-derived lipid mobilization during zebrafish development, whereas *esyt3* is more closely associated with intracellular lipid transfer and membrane lipid homeostasis [50–52]. Such broad repression indicates that OPE induces a collapse of the lipid transport network rather than disrupting a single pathway. This multigenic suppression plausibly explains the lipid accumulation and developmental delays observed in both zebrafish embryos and *Daphnia magna*, establishing a clear link between molecular disruption and phenotypic toxicity [53–55]. From an ecological perspective, impaired lipid transport can retard growth and development in aquatic organisms, thereby reducing their ability to escape predation or compete for food resources. Such physiological deficits may, in turn, alter predator–prey dynamics and disrupt energy flow within freshwater ecosystems [56–58].

Despite its strengths, this study has several limitations. First, we selected O_2_ plasma treatment as a rapid, controllable, and reproducible approach for introducing oxygen-containing functional groups under well-defined conditions [59, 60]. We explicitly acknowledge that this laboratory model does not capture the multi-factorial complexity of natural, long-term environmental weathering, which includes mechanical abrasion and microbial biofouling. Accordingly, the term “oxidized polyethylene” in this study refers to plasma-induced surface oxidative modification rather than extensive bulk degradation. Nevertheless, this standardized acceleration model was methodologically advantageous for this study’s scope, as it allowed us to establish a clear cause-and-effect relationship between chemical oxidation and lipid metabolic disruption without interference from external environmental factors. Future studies incorporating naturally weathered microplastics gathered from the field will be essential to validate these mechanistic findings under real-world scenarios.

## Conclusions

In conclusion, this study demonstrates that surface-localized chemical oxidation can markedly amplify the ecotoxicological impacts of polyethylene microplastics. While our laboratory-controlled oxygen plasma treatment represents an accelerated surface oxidation model rather than a replication of multi-factorial natural weathering, it successfully isolates chemical oxidation as a primary driver of enhanced toxicity. Mechanistically, our findings suggest that this oxidative modification can increase the biological activity of polyethylene particles, facilitating their penetration through biological barriers and exacerbating lipid metabolic disruption in a dual-species in vivo framework. Specifically, the significant down-regulation of lipid transport pathways, particularly characterized by the suppression of *mttp* and apolipoprotein-related transcripts, underscores a conserved molecular mechanism of microplastic-induced metabolic disruption across aquatic taxa.

These findings provide a critical mechanistic foundation by demonstrating how structural aging alters the biological interactions and toxicological behavior of microplastics. Given that microplastics in the environment can undergo diverse physicochemical transformations, our work emphasizes the vital necessity of moving beyond pristine polymers in ecotoxicological frameworks. Incorporating chemical aging states and surface functional variations into future safety evaluations will be essential for establishing more precise, mechanism-based environmental risk assessments.

## Supplementary Information

Supplementary Material 1.

## Data Availability

The data that support the findings of this study are available from the corresponding author upon reasonable request. RNA sequencing data using zebrafish embryos utilized for this study is available at Gene Expression Omnibus (GEO, https://www.ncbi.nlm.nih.gov/geo/) under accession number GSE281865 and GSE304012.

## Abbreviations

MPs: Microplastics
PE: Polyethylene
OPE: Oxidized polyethylene
OMPs: Oxidized microplastics
ROS: Reactive oxygen species
DLS: Dynamic light scattering
SEM: Scanning electron microscopy
FTIR: Fourier-transform infrared spectroscopy
GI: Gastrointestinal
GO: Gene Ontology
DEG: Differentially expressed gene
PCA: Principal component analysis
PPI: Protein–protein interaction
RNA-seq: RNA sequencing
qPCR: Quantitative polymerase chain reaction
PFA: Paraformaldehyde
PBS: Phosphate-buffered saline
TDW: Triple-distilled water
hpf: Hours post-fertilization
MTTP: Microsomal triglyceride transfer protein
VLDL: Very-low-density lipoprotein
GEO: Gene Expression Omnibus
OECD: Organisation for Economic Co-operation and Development
FPKM: Fragments per kilobase of transcript per million mapped reads
RT: Room temperature
