# Supplementary figures and images for: Oxidative aging facilitates biological barrier penetration of polyethylene microplastics, amplifying systemic lipotoxicity in aquatic species
Source: Part Fibre Toxicol. 2026 Jun 7;23:36. doi: 10.1186/s12989-026-00689-2 (PMC13285457; doi:10.1186/s12989-026-00689-2)

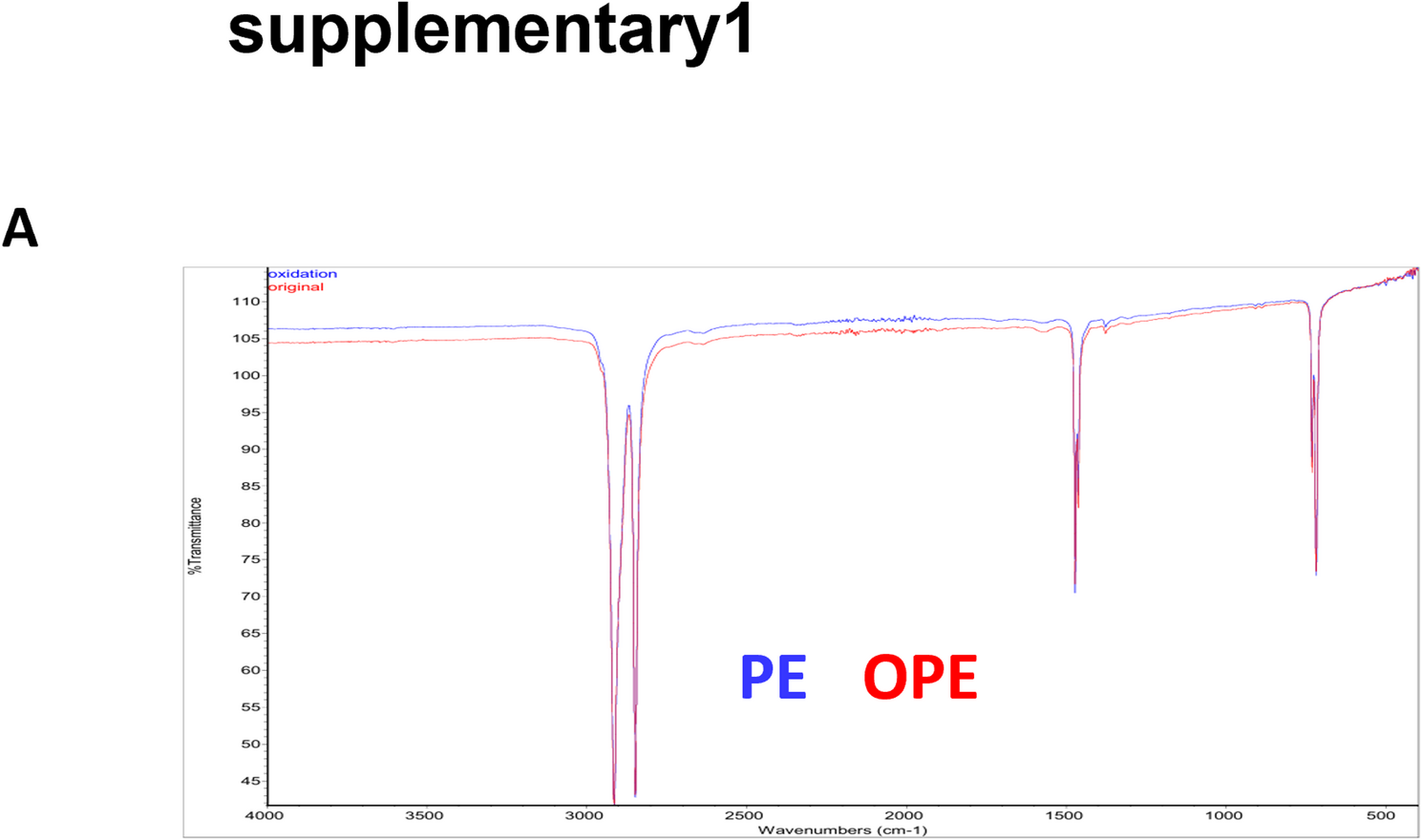

Supplement: Supplementary file 1 — Supplementary Material 1. [file 12989_2026_689_MOESM1_ESM.png]
